# Dynamics of chemosensitivity and chromosomal instability in recurrent glioblastoma

**DOI:** 10.1038/sj.bjc.6603652

**Published:** 2007-03-06

**Authors:** S Spiegl-Kreinecker, C Pirker, C Marosi, J Buchroithner, J Pichler, R Silye, J Fischer, M Micksche, W Berger

**Affiliations:** 1 Departments of Neurosurgery, Wagner Jauregg Hospital, Linz, Austria; 2 Clinical Division of Oncology, Department of Medicine I, Medical University Vienna, Vienna, Austria; 3 Department of Internal Medicine, Wagner Jauregg Hospital, Linz, Austria; 4 Institute of Pathology, Wagner Jauregg Hospital, Linz, Austria; 5 Institute of Cancer Research, Department of Medicine I, Medical University Vienna, Vienna, Austria

**Keywords:** Recurrent glioblastoma, chemosensitivity, chromosomal instability, temozolomide, MGMT

## Abstract

Glioblastoma multiforme is characterised by invasive growth and frequent recurrence. Here, we have analysed chromosomal changes in comparison to tumour cell aggressiveness and chemosensitivity of three cell lines established from a primary tumour and consecutive recurrences (BTL1 to BTL3) of a long-term surviving glioblastoma patient together with paraffin-embedded materials of five further cases with recurrent disease. Following surgery, the BTL patient progressed under irradiation/ lomustine but responded to temozolomide after re-operation to temozolomide. The primary tumour -derived BTL1 cells showed chromosomal imbalances typical of highly aggressive glioblastomas. Interestingly, BTL2 cells established from the first recurrence developed under therapy showed signs of enhanced chromosomal instability. In contrast, BTL3 cells from the second recurrence resembled a less aggressive subclone of the primary tumour. Although BTL2 cells exhibited a highly aggressive phenotype, BTL3 cells were characterised by reduced proliferative and migratory potential. Despite persistent methylation of the *O*^*6*^-methylguanine-DNA methyltransferase promoter, BTL3 cells exhibited the highest temozolomide sensitivity. A comparable situation was found in two out of five glioblastoma patients, both characterised by enhanced survival time, who also relapsed after surgery/chemotherapy with less aggressive recurrences. Taken together, our data suggest that pretreated glioblastoma patients may relapse with highly chemosensitive tumours confirming the feasibility of temozolomide treatment even in case of repeated recurrence.

Malignant transformation and progression is thought to be based on the stepwise accumulation of genetic changes that confer growth and survival advantages to (pre)malignant cells. Consequently, solid tumours at diagnosis are generally characterised by complex karyotypic alterations and an increasing number of chromosomal aberrations have been shown to be associated with tumour development and progression (reviewed by Albertson *et al* (2003)). This clonal evolution in combination with chromosomal instability may result in the development of variant subclones within one tumour harbouring different sets of genetic changes.

Human malignant gliomas represent the most common primary malignant brain tumour comprising astrocytomas, oligodendrogliomas, ependymomas, and tumours of the coroid plexus. The by far most frequent histological entity is glioblastoma (astrocytoma WHO grade IV), typically composed of different cell types displaying a wide spectrum of heterogeneity regarding morphology (referred to by the term ‘multiforme’), biological aggressiveness, invasive potential, as well as treatment sensitivity (Mao and Hamoudi, 2000; Zhu and Parada, 2002). Glioblastomas arise either *de novo* without evidence of an antecedent lesion (primary glioblastoma) or through an anaplastic progression from a lower-grade astrocytoma (secondary glioblastoma). Genetic studies revealed that there are different distinct pathways involved in the development of these lesions. Although in primary glioblastoma loss of the tumour suppressors p16/Cdkn2/Ink4 and PTEN as well as amplifications in EGFR and HDM2 are frequent, secondary glioblastomas lose p53 already at the stage of low-grade astrocytoma (Mao and Hamoudi, 2000; Zhu and Parada, 2002).

With respect to chromosomal aberrations deletions of 1p, 9p, 10, 13q, 17p, 19q, and 22q, and gains of chromosome 7 (Liu *et al*, 1997; Rasheed *et al*, 1997; Duerr *et al*, 1998; Smith and Jenkins, 2000) are the most common in gliomas. Several genes have been identified to be associated with tumorigenesis and anaplastic progression of glioblastoma subgroups including, besides the already mentioned p16/Cdkn2/Ink4, EGFR, PTEN, p53, and HDM2 also for example cdk4, cyclin D1, PDGFR*α*, k-ras, N-myc, gli, c-myc, and myb (Mao and Hamoudi, 2000; Zhu and Parada, 2002).

Despite intensive research regarding the genetics and biology, the prognosis of glioblastoma patients is still poor. Even in case of maximal therapy, median survival of glioblastoma patients remains low and ranges between 10 and 12 months, only (Scott *et al*, 1998). Thus, a more profound knowledge of the molecular background and cell biological behaviour of this cancer is inevitable to develop appropriate treatment strategies (Buckner, 2003).

The current standard of care for patients with high-grade malignant glioma used to be resection followed by radiotherapy (RT). However, the use of adjuvant and/or concomitant chemotherapy and the standard of care at first relapse are still under debate (van den Bent *et al*, 2006). Chemotherapeutic treatment success in brain tumours is mainly limited by the blood–brain barrier and the expression of chemoresistance-related proteins like P-glycoprotein (P-gp), multidrug resistance protein 1 (MRP1), and major vault protein (MVP) also known as lung resistance protein (LRP) (Berger *et al*, 2001; Bredel, 2001; Spiegl-Kreinecker *et al*, 2002). Based on their ability to circumvent the blood–brain barrier, alkylating agents represent the gold standard in the treatment of malignant gliomas. Besides nitrosoureas, temozolomide is the most promising drug in the current treatment of patients with malignant gliomas. Temozolomide has demonstrated a good tissue distribution, including penetration of the blood–brain barrier and the cerebrospinal fluid (DeAngelis, 2003). Recently, a significant survival benefit for glioblastoma patients treated with temozolomide combined with RT was shown (Stupp *et al*, 2005). However, protection against the toxic effect of alkylating agents might rely on the activity of *O*^6^-methylguanine-DNA methyltransferase (MGMT) (Harris and Margison, 1993) a key enzyme in the DNA repair network.

In this study, we present, to the best of our knowledge, a unique glioblastoma cell model consisting of one primary tumour- and two recurrences-derived permanent cell lines from a single patient (BTL). Chromosomal changes as well as cell-biological behaviour and treatment responsiveness were compared to the respective clinical observations and to five further patients with recurrent glioblastoma (GL1–5). We demonstrate that even in case of repeated recurrence and heavy pretreatment with chemotherapy – believed to be often associated with acquired chemotherapy resistance (Bredel, 2001) – glioblastoma patients may relapse with less aggressive tumours highly responsive to chemotherapy.

## MATERIALS AND METHODS

### Patient characteristics and clinical course of disease

BTL cells were derived from a 51-year-old woman who underwent surgery of glioblastoma within the right frontal lobe at the Department of Neurosurgery, Wagner-Jauregg-Hospital, Linz, Austria. The clinical course of disease and treatment is depicted in Table 1. Post–operative before RT, the patient received one cycle lomustine (CCNU) (100 mg m^−2^ body surface area). Radiotherapy started 6 weeks after surgery, but had to be ceased incomplete (4000 cgy) because of tumour progression. The patient was re-operated 3 months after resection of the primary tumour and afterwards received seven cycles temozolomide (200 mg day^−1^ 1–5 every 28 days). After the third cycle, reduction of the tumour bulk was observed by magnetic resonance imaging (MRI), which was confirmed as partial regression after the seventh cycle. Tumour regression was accompanied by continual improvement of performance status. After 8 months, a second recurrence, indicated by sudden worsening of performance status, was confirmed by MRI. Consequently, the patient underwent re-surgery 17.5 months after the first relapse but refused further chemotherapy. After 3 months, MRI confirmed tumour progression leading to disease-related death 27 months after initial diagnosis.

Formalin-fixed, paraffin-embedded tissues of primary tumours and recurrences derived from additional five glioblastoma patients (GL1–5) with recurrent disease were obtained from the Institute of Pathology, Wagner Jauregg Hospital, Linz. Therapy response and overall survival times are depicted in Table 3. All patients gave informed consent.

### Establishment of tumour cell cultures

Primary cell cultures were established from all three surgery specimens (BTL1, BTL2, and BTL3) and were used at passage numbers 5–15 for analysis. Patches from non-necrotic parts of the tumour were obtained during surgery, minced mechanically and transferred into culture flasks, containing growth medium (RPMI 1640, 20% fetal calf serum (FCS), 1% glutamine, 1% penicillin/streptomycine; PAA Laboratories, Linz, Austria). After the third passage, cells were grown in growth medium with 7% FCS and 1% glutamine and without antibiotics. Three to five culture flasks were set up from each surgery specimen. Cell cultures were then pooled during the first passages. All cell cultures were periodically checked for *Mycoplasma* contamination (Mycoplasma Stain Kit; Sigma, St Louis, MO, USA).

### Cytogenetic analyses

Microdissection of paraffin-embedded materials was carried out using the Leica AS LMD Laser Microdissection System (Cambridge, UK). Preparation of genomic DNA was performed as described previously (Pirker *et al*, 2004). Comparative genomic hybridisation (CGH), fluorescence *in situ* hybridisation (FISH) and CDD banding were performed as described previously (Pirker *et al*, 2004). For DNA amplification, linker–adapter PCR was used as described (Pirker *et al*, 2004). For the detection of the EGFR locus, the BAC clone RP11-339F13 supplied by Pieter De Jong (Oakland, CA, USA) was used.

### Growth curves

A total of 5 × 10^4^ cells well^−1^ were seeded into six-well plates. Every 48 h cells were collected and counted in 0.2% trypan blue solution, whereas medium changes were performed for the remaining samples.

### Scratch assay

Cells (2 × 10^5^ well^−1^) were plated in a six-well plate. When the cells had reached about 90% confluence, the monolayer was wounded by setting an ‘X’-shaped scratch with a sterile plastic tip. Several areas of the scratch were documented by photomicrographs and followed for up to 44 h. The width of the gap was measured at several time points with a Leica TE100 microscope equipped with a CCD camera (Cambridge, UK) and the gap width was measured using Metamorph 6.1 software (Universal Imaging Corp., Biocompare).

### Bisulphite modification and MSP

Methylation-specific PCR (MSP) exploits the effect of sodium bisulphite on DNA, which efficiently converts unmethylated cytosine to uracil but which leaves methylated cytosine unchanged. DNA from cultured cells was extracted with QIAmp DNA Blood Mini Kit (Qiagen, Valencia, CA, USA) and 1 μg of genomic DNA was denatured with NaOH (3 M). Following denaturation, 10 mM hydrochinone (Sigma) and 3 M sodium-bisulphite (Sigma) were added and DNA modification was performed at 50°C for 16 h. DNA samples were then purified with the Wizard DNA purification resin (Promega, Mannheim, Germany) followed by ethanol precipitation. Methylation-specific PCR was performed with primers specific for either methylated or modified unmethylated DNA, as described previously (Esteller *et al*, 1999). For PCR a 25 *μ*l mix consisting of DNA, specific primers and HotStarTaqMasterMix (1.25 U HotStarTaq DNA Polymerase; 1 × PCR buffer; 100 μM of each dNTP; Qiagen) was prepared and amplification was carried out in the iCycler Thermal Cycler (BioRad, Hercules, CA, USA) starting with an initial activation step at 95°C for 15 min. Thirty-five cycles were performed with an annealing temperature of 66°C. DNA from melanoma metastasis primary cell cultures served as control for methylated as well as unmethylated MGMT promoter. A total of 10 *μ*l of each PCR reaction were loaded onto a 6% polyacrylamide gel and ethidium bromide-stained gels were visualised under UV illumination (ChemiDoc; BioRad). Expression levels were quantified by Quantity One Quantitation software (BioRad) and calculated relatively to the indicated controls.

### RT–PCR

mRNA expression of the chemoresistance-related proteins MGMT, P-gp, MVP, and MRP1 was determined by RT–PCR analysis. Total cellular RNA was extracted and RT–PCR performed with One Step RT–PCR Kit (Qiagen) using oligonucleotide primer sets specific for MGMT (sense 5′-CCTGGCTGAATGCCTATTTC-3′; antisense 5′-CAGCTTCCATAACACCTGTCT-3′; product size: 116 bp), P-gp (sense 5′-CCCATCATTGCAATAGCAGG-3′; antisense 5′-GTTCAAACTTCTGCTCCTGA-3′; 170 bp), MVP (sense 5′-TTCTGGATTTGGTGGACGC-3′; antisense 5′-MVP antisense 5′-ACTTCTCTCCCTTGACCA-3′; 284 bp) and MRP1 (sense 5′-GGACCTGGACTTCGTTCTCA-3′; MRP1 antisense 5′-CGTCCAGACTTCTTCATCCG-3′; 291 bp). GAPDH was amplified (358 bp) as described in (Berger *et al*, 2000) as a housekeeping gene. For semiquantitative evaluation, 30 cycles were chosen for MGMT, P-gp, MVP, and MRP1 and 22 cycles for GAPDH. Amplification was performed in a thermal cycler (iCycler; Bio-Rad) under the following cycle conditions: 94°C for 30s; 56°C for 40 s; 72°C for 40 s. Amplification products were separated by acrylamide gel electrophoresis, stained with ethidium bromide and quantified by scanning densitometry (Chemi Doc, Quantity One Quantitation software, Bio-Rad, Hercules, CA, USA). Expression levels (arbitrary units) were calculated relatively to GAPDH mRNA amplified concomitantly.

### Western blot

Protein expression was determined by immunoblot analysis. Total protein extracts (determination of MGMT and MVP) as well as preparation of crude membrane extracts (P-gp, MRP1) were prepared as described previously (Berger *et al*, 1997; Berger *et al*, 2000) and protein concentrations measured with BCA Protein Assay (Pierce, Rockford, IL, USA). Proteins were separated by SDS–PAGE and transferred onto nitrocellulose membranes (Hybond ECL, Amersham, Aylesbury, UK). Blots were probed with monoclonal antibodies against MGMT (Dako, Glostrup, Denmark), MVP (Transduction Laboratories, Lexington, KY, USA), P-gp (C219; Centocor, Malvern, PA, USA) and MRP1 (MRPr1; Alexis Biochemicals, San Diego, CA, USA). Visualisation and quantification were performed using the ChemiDoc System (BioRad, Hercules, CA, USA). In previous studies, multiple glioblastoma cell lines established in our laboratory were extensively characterised for their expression of drug resistance genes (Berger *et al*, 2001; Bredel, 2001; Spiegl-Kreinecker *et al*, 2002). Respective protein extracts of the following cell lines have been used as positive controls in Western blot analysis: for MGMT GL80, for MVP GL54, for MRP1 GL52 and for P-gp YT-BO.

### Chemosensitivity testing

The impact of the investigated drugs on cell proliferation and viability was tested by establishing whole dose–response curves using an MTT-based survival assay (EZ4U, Easy-for-you, Biomedica, Vienna, Austria). Cells in logarithmic growth phase were seeded in triplicates into three 96-well plates (3 or 1.5 × 10^3^ 100 *μ*l well^−1^) and incubated for 24 h at 37°C. All drugs were applicated for 72 h or 5 days continuous exposure. The surviving proportions of cells as compared to the untreated controls were determined using EZ4U according to the instructions of the manufacturer. Experiments were repeated three times and IC_50_ values were calculated from whole dose–response curves.

Additionally, the impact of temozolomide on ^3^H-thymidine incorporation was determined in BTL cells. Cells (4 × 10^3^ per 100 *μ*l well^−1^) were seeded into a 96-well plate and after 24 h exposed for 48 h to 50–1500 *μ*M temozolomide. After the exposure culture medium was replaced for 1 h by a 2 nM ^3^H-thymidine solution (diluted in full culture medium, radioactivity: 25 Ci mM^−1^). Afterwards cells were washed three times with PBS, cell lysates prepared and radioactivity was determined as described (Berger *et al*, 1994). Experiments were carried out in triplicates and results (means of two experiments) are expressed relatively to the untreated controls set arbitrarily as 1.

In order to determine the cytotoxic activity of temozolomide, Hoechst 33258/propidium iodide (PI) co-staining (both from Sigma) to determine the number of viable and apoptotic/dead cells was performed as described (Grusch *et al*, 2001). Cells in logarithmic growth phase were seeded in triplicates into 96-well plates (1 × 10^3^ per 100 *μ*l well^−1^) and incubated for 24 h at 37°C. Temozolomide was applicated for 5 days of continuous exposure. Hoechst 33258 and PI were added directly to the culture medium to final concentrations of 5 and 2 *μ*g ml^−1^, respectively. After an incubation period of 1 h at 37°C the cells were examined on a Zeiss Axiovert 35 fluorescence microscope with DAPI filters, and viable (Hoechst-positive, PI-negative) and dead (Hoechst-positive, PI-positive)/apoptotic (Hoechst-positive with condensed/fragmented chromatin, PI-negative) cells were counted.

### Drugs

Temozolomide and liposomal doxorubicin (L-DOX) were kindly provided by AESCA Pharma (Traiskirchen, Austria). All other drugs used were obtained from Sigma (St Louis, MO, USA).

Cytotoxic effects were analysed for daunomycin (9–180 nM), etoposide (VP16; 0.5–25 *μ*M), cisplatin (CDDP; 0.25–5 *μ*M), doxorubicin (DOX; 10–150 nM), L-DOX (10–150 nM), bleomycin (BLEO 1–100 mU ml^−1^), carmustine (BCNU; 50–600 *μ*M), and temozolomide (TMZ 100–1500 *μ*M). Drugs were dissolved in physiological saline (DM, DOX), DMSO (VP16, TMZ), ethanol (BCNU), and aqua bidest (BLEO, CDDP). L-DOX was provided as sterile solution ready for use.

## RESULTS

### Cytogenetic analyses of BTL1-3 cells show changes typical for glioblastoma multiforme

**Figure 1:**
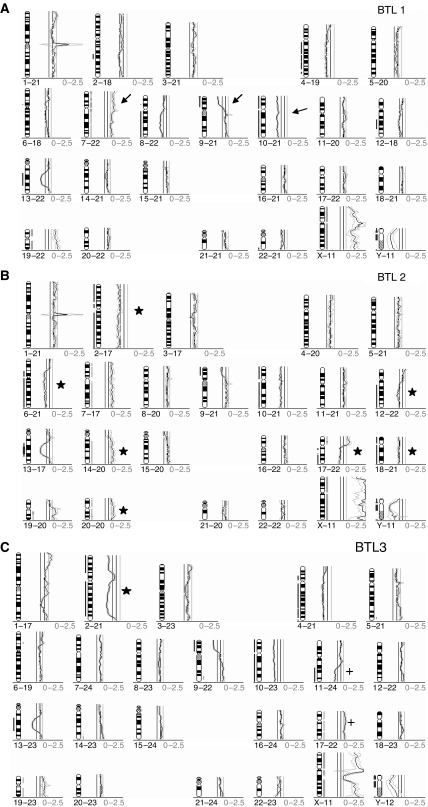
CGH analysis profiles (for interpretation see Pirker *et al*, 2003) of (**A**) primary tumour-derived BTL1, (**B**) first recurrence-derived BTL2, and (**C**) second recurrence-derived BTL3 cells. Arrows in (**A**) indicate typical changes for glioblastoma multiforme. Asterisks in (**B**) indicate additional chromosomal aberrations in BTL2 as compared to BTL1. In (**C**) the asterisk indicates an aberration present also in BTL2 but not BTL1. Crosses in (**C**) represent changes unique to BTL3.

**Figure 2:**
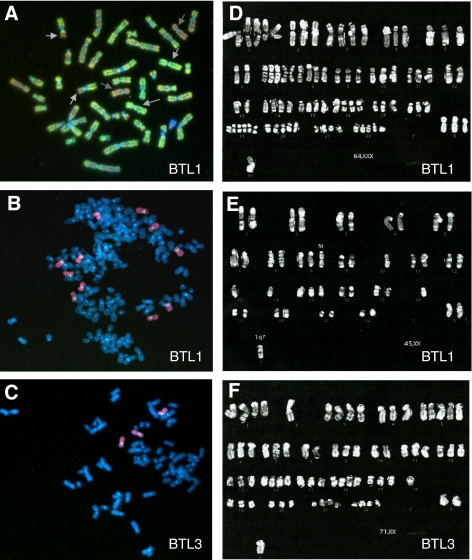
Cytogenetic analysis of the BTL glioblastoma cell model. Representative CGH (**A**), FISH (**B**, **C**), and CDD banding (**D**–**F**) analyses of the indicated cell lines are shown. Arrows in (**A**) indicate typical changes for glioblastoma multiforme. For FISH analysis (**B**, **C**) a paint for whole chromosome 7 (red) and a BAC clone for the EGFR locus (green) were used. (**D** and **E**) show representative karyograms of the two subclones detected in BTL1.

Three cell lines termed BTL1, BTL2, and BTL3 were established from consecutive surgeries of a primary tumour and two recurrences of a long-term surviving glioblastoma patient. The respective course of disease, therapies, and the time points of surgery are summarised in Table 1. Chromosomal changes in the cell lines were analysed by CGH and classical cytogenetics. Figure 1A–C present the CGH profiles of all three cell cultures. The primary tumour-derived cell line BTL1 contained chromosomal changes typical for glioblastoma, for example, gain of chromosome 7, loss of 9p, as well as loss of chromosome 10 (indicated by arrows in Figure 1A). A respective CGH metaphase for BTL1 is given in Figure 2A. BTL2 cells, derived from the first recurrence progressing during RT and chemotherapy, showed numerous additional chromosomal changes as compared to BTL1 (asterisks in Figure 1B). Some of these aberrations (losses at chromosomes 2, 6q, and 12q) reached only borderline significance in BTL1 and were distinctly more pronounced in BTL2, whereas other changes (gains at chromosomes 14, 17p, and 20, losses at chromosomes 17q and 18) were completely new in BTL2. This increase in genomic aberrations reflects a high degree of chromosomal instability of BTL2 cells. Unexpectedly, CGH analysis of BTL3 cells, derived from a second recurrence resected 15 months later, revealed a less aberrant chromosomal pattern with loss of most changes present in BTL2 (a remaining change is shown as asterisk in Figure 1C) and only very few new changes (crosses in Figure 1C). Interestingly, chromosome 7 amplifications present in BTL1 and BTL2 were completely lost in BTL3 cells. Conventional cytogenetics (Figure 2D–F), as well as FISH analysis of BTL cells (Figure 2B and C), widely confirmed the results obtained by CGH. The CDD banding of tumour metaphase chromosomes revealed the existence of at least two different subclones in BTL1 cells. The predominant subclone showed a highly aberrant, nearly tetraploid karyotype with additional copies of chromosome 7 (Figure 2D), whereas the second subclone displayed a less aberrant, nearly diploid karyotype with only two copies of chromosome 7 (Figure 2E). FISH analysis with chromosome paint for chromosome 7 and a specific BAC clone against the EGFR gene locus confirmed these results (Figure 2B). The highly aberrant and instable karyotype of BTL2 cells was confirmed by CDD banding and FISH (data not shown). CDD banding as well as FISH data for BTL3 revealed a mainly triploid karyotype and, corresponding with CGH results, three copies of chromosome 7 (Figure 2C and F).

### CGH analyses of glioblastoma multiforme primary tumours and recurrences

In addition to the BTL cell model, paraffin-derived materials from the primary tumour and one or two recurrences of five further glioblastoma patients were available for analysis. The numbers of chromosomal changes in these tumour samples were compared to chemotherapy responses and overall survival (Table 3). Tumours of patients GL1, GL2, and GL3 showed increased chromosomal aberrations during disease progression, therapy resistance, and relatively short overall survival (mean of 17.67 months). In contrast, patients GL4 and GL5 displayed distinctly reduced chromosomal instability and in case of GL5 therapy sensitivity in recurrent tumour thus resembling the *in vitro* BTL cell model. Interestingly, all three respective patients showed remarkable long overall survival (mean of 33 months, patient GL5 still alive at 26 months) although GL4 had refused chemotherapy at recurrence. These observations again indicate that chemosensitive glioblastoma patients may relapse with a less aggressive tumour subclone corresponding to long-term patient survival.

### Growth and migration potential

**Figure 3:**
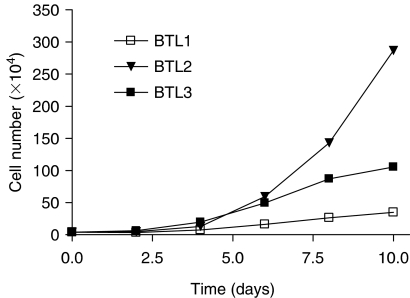
*In vitro* growth dynamics of the BTL glioblastoma cell model. Growth curves for the indicated cell lines were established as described in Material and Methods. One of three experiments with comparable results is shown.

In parallel to the chromosomal changes, several determinants of tumour cell malignancy were analysed in BTL cells. *In vitro* cell growth potentials were analysed by establishment of growth curves (Figure 3). Whereas the primary tumour -derived BTL1 cells were relatively slow-growing, BTL2 cells showed significantly increased growth potential especially at enhanced cell density. In contrast, BTL3 cells displayed reduced cell proliferation as compared to BTL2 and trended to enter plateau stage at higher density.

**Figure 4:**
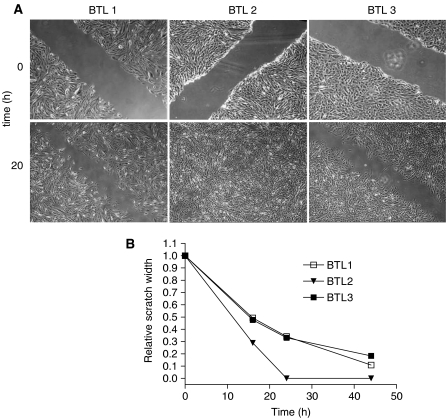
Migratory potential of the BTL glioblastoma cell model. Scratch assays were performed as described in Materials and Methods. (**A**) Representative photomicrographs taken at the indicated time points are shown. (**B**) To determine the relative closure of the gap, photomicrographs as shown in (**A**) were taken at different time points and measured using MetaMorph 6.1 software. The gaps’ widths immediately after wounding were arbitrarily set as 1. At least three experiments with comparable results were performed.

Additional to cell growth, the migratory potential of all BTL cell lines was assessed by monolayer scratch assays. Figure 4A shows photomicrographs of representative scratches in BTL cell monolayers immediately after wounding and following 20 h of incubation. Figure 4B summarises graphically the results from several analysed time points. Although BTL3 cells had completely closed the wound already within 20 h, for both BTL1 and BTL3 the scratch was still visible even after 44 h of incubation. At these late time points, the parental BTL1 cell line exhibited a slightly higher migratory potential as compared to the second recurrence-derived BTL3 cells that were unable to close the wound even by 72 h (data not shown).

### Sensitivity against chemotherapeutic interventions

**Figure 5:**
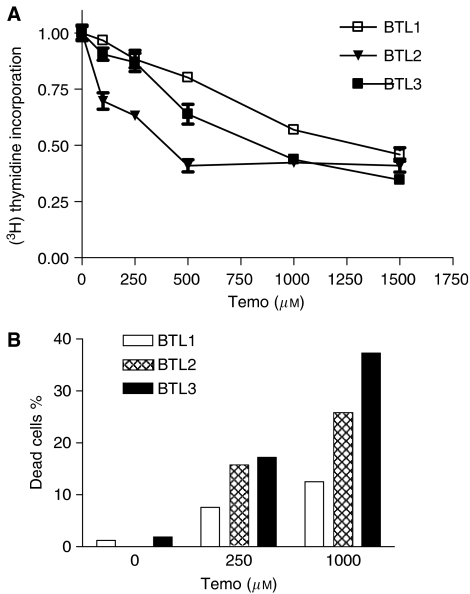
Antiproliferative and cytotoxic effects of temozolomide against BTL cells. (**A**) The indicated BTL cell lines were exposed to increasing temozolomide concentrations for 48 h and the rate of DNA synthesis was determined by ^3^H-thymidine incorporation. Data are given relatively to the untreated controls set as 1. Means of two experiments in triplicates are shown. (**B**) BTL cells were exposed for 5 days to the indicated temozolomide concentrations and the percentage of apoptotic/dead cells was determined by Hoechst 33258 and PI staining as described in Materials and Methods. One representative example out of three experiments delivering comparable results is shown.

Chemosensitivity of BTL cell lines against several anticancer agents was established by MTT-based survival assays (Table 2). Using a 72 h drug exposure, an increasing sensitivity against temozolomide within the BTL cell model was detected by the vitality assay. Thus, BTL3 cells, derived from a recurrence 9 months after successful temozolomide treatment (compare Table 1), exhibited the highest temozolomide sensitivity. With respect to a 72 h continuous drug exposure, activity of the second alkylating agent BCNU revealed an opposite resistance pattern with the lowest IC_50_ value in BTL1 cells. Resembling temozolomide, also, the sensitivity against anthracyclines tended to be enhanced in the recurrence-derived sublines with the strongest differences observed in case of doxorubicin. As characteristic of glioblastoma cells (Bredel, 2001), all BTL sublines were resistant against VP-16, bleomycin, and CDDP. Extending the exposure time in the case of temozolomide to 5 days confirmed the lowest temozolomide sensitivity for the primary tumour -derived cell line BTL1 (IC_50_: 605.8 *μ*M), whereas at that exposure time BTL2 cells showed the highest treatment response (IC_50_: 158.3 *μ*M). The higher temozolomide sensitivity of BTL2 cells with increasing exposure time might be explained by the enhanced proliferation of this cell line at higher densities (compare Figure 3). To test this hypothesis, we analysed the impact of a 48 h temozolomide exposure on DNA synthesis of BTL cells seeded at enhanced density by ^3^H-thymidine incorporation (Figure 5A). Indeed, temozolomide treatment reduced DNA synthesis of BTL2 cells significantly stronger as compared with BTL3, whereas BTL1 cells showed the weakest response. In order to explain the higher temozolomide sensitivity of BTL3 cells in the 72 h survival assay (compare Table 2), we additionally analysed the cytotoxicity of temozolomide against BTL cells at a 5-days exposure time (Figure 5B). Both recurrence-derived cell lines demonstrated a significantly enhanced percentage of apoptotic/dead cells as compared to parental BTL1 cells with BTL3 showing the highest response to the cytotoxic impact of temozolomide.

### Gene expression of chemoresistance-related proteins

**Figure 6:**
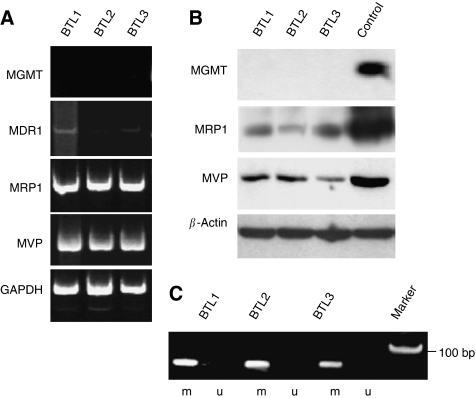
Expression of chemoresistance-related genes in BTL glioblastoma cells. mRNA (**A**) and protein (**B**) levels encoded by the indicated chemoresistance genes were determined by RT–PCR and Western blot, respectively. (**C**) Methylation of the MGMT promoter was detected by methylation-specific PCR and a representative experiment is shown (m=methylated; u=unmethylated). Protein extracts of the glioblastoma cell lines GL80, GL54, and GL52 were used as positive controls for MGMT, MVP, and MRP1, respectively.

Expressions of the DNA repair enzyme MGMT as well as the chemotherapy resistance markers P-gp, encoded by the mdr1 gene, MRP1, and MVP were detected by RT–PCR (Figure 6A) and Western blots (Figure 6B). All the three cell lines lacked MGMT mRNA and protein expression. Correspondingly, methylation of the MGMT gene promoter was persistently detected in all three cell lines (Figure 6C). Only very low amounts of mdr1 mRNA were detectable by RT–PCR in BTL1 cells, which was even decreasing from BTL2 to BTL3 (Figure 6B). In contrast, P-gp expression was under the detection limit in all three sublines (data not shown). Whereas MRP1 expression was constantly expressed in all cell lines, MVP levels decreased continuously during disease progression.

## DISCUSSION

Multiple genetic alterations are generally found in solid tumours and an increasing complexity of chromosomal aberrations is typically associated with tumour progression (Albertson *et al*, 2003). This implies that chromosomal instability can act as a driving force for tumorigenesis, leading to tumour cell immortalisation and the acquisition of new subclones with features of enhanced aggressiveness. Based on the assumption that gliomas are clonal tumours (Bonneau and Longy, 2000), both shared genetic changes as well as the appearance of additional alterations during disease progression are expected to be found in glioblastomas and their recurrences (Fujisawa *et al*, 1999; Saxena *et al*, 1999). Similarly, acquisition of therapy resistance is believed to depend on the selection of subclones exhibiting activation of the respective resistance mechanisms (Bredel, 2001). In this study, we have analysed the dynamics of chromosomal instability and chemosensitivity during glioblastoma progression using a unique cell model comprising three cell lines derived from surgery specimens of a long-term surviving glioblastoma patient. Cell cultures were obtained from the primary tumour before therapy and two consecutive recurrences resected during and 9 months after termination of RT- and/or chemotherapy, respectively (Table 1). The cell biological analyses of these cell models argue against the hypothesis of a linear increase of tumour aggressiveness and therapy resistance during disease progression and reveal that even late glioblastoma recurrences can represent relatively inaggressive and highly chemosensitive tumour cell subclones. Although these data are derived from only one cell model, the respective conclusions were also corroborated by the analysis of paraffin-embedded material obtained from primary tumours together with the corresponding recurrences of five additional glioblastoma patients. Although in the three therapy-resistant cases chromosomal aberrations increased during disease progression, two patients developed a recurrence with a less aberrant genotype. Paralleling the BTL cell model, one of these patients (GL5) responded to chemotherapy after relapse, whereas the other patient (GL4) unfortunately refused further chemotherapy. All three patients recurring with less aberrant genotypes showed a remarkably long survival time. These data point towards general differences in the dynamics of chromosomal instability and chemosensitivity in short- and long-term surviving glioblastoma patients. However, it has to be kept in mind that the current study only involves one cell model and five additional glioblastoma patients. Thus, further studies, including extended patient numbers and, if possible, further cell models, are necessary to confirm our observations.

Intratumoral heterogeneity of glioblastoma multiforme has been mostly described with regard to histopathological features; however, also the presence of genetically different cell clones in glioblastoma at diagnosis has been occasionally reported (Hulsebos *et al*, 2004). Correspondingly, cytogenetic analysis of our primary tumor-derived BTL1 cell line demonstrated the presence of at least two cell subclones with different chromosomal aberrations. Although one of them exhibited strong chromosomal instability and alterations associated with high aggressiveness, CDD banding revealed the existence of a second, less rearranged and nearly diploid subclone. Interestingly, these subclones resembled in several aspects those cell lines established from the first and second recurrence, namely BTL2 and BTL3 cells, respectively.

In CGH analysis, the primary tumor- and the first recurrence-derived cell lines BTL1 and BTL2 exhibited several alterations characteristic of highly malignant gliomas, including loss of chromosome 10q, with the PTEN and the MGMT gene locus on 10q23.3 and 10q26, respectively, loss of material from chromosome 9p, with the CDKN2A locus on 9p16, and gain of chromosome 7 resulting in amplifications at the epidermal growth factor receptor (EGFR) locus on 7p12 (Inda *et al*, 2003). Interestingly, CGH and FISH analyses revealed that BTL3, the second recurrence-derived cell line, harboured neither any gains of chromosome 7 nor additional EGFR copy numbers. Moreover, BTL3 cells demonstrated reduced proliferative and migratory potential. This suggests loss of several features of glioblastoma aggressiveness in BTL3 probably based on lower EGFR-mediated signals (Aghi *et al*, 2005; Kang *et al*, 2005; Lamszus *et al*, 2005). Loss of chromosome 7 copy numbers was also observed in the recurrent tumour of GL5, showing only a borderline gain of chromosome 7, whereas the primary tumour displayed chromosome 7 amplification (data not shown). Accordingly, EGFR amplification has been shown to be a negative prognostic factor for overall survival of glioblastoma patients and to correlate with shorter time to tumour progression (Schlegel *et al*, 1994a, 1994b; Zhu *et al*, 1996; Etienne *et al*, 1998). Also gain of chromosome 7 together with loss of chromosome 10, the most frequently found combined genetic alterations in glioblastomas, are associated with poor prognosis and short survival (Wiltshire *et al*, 2000; Ohgaki *et al*, 2004; Nigro *et al*, 2005).

With respect to BTL2 cells, the cytogenetic data suggest that the first recurrence is derived from the more aggressive subclone of the primary tumour, which might be reflected by chromosomal changes that are borderline in BTL1 but reach significance in BTL2 (losses in chromosomes 2, 6q, and 12q). The additional new changes in BTL2 might be caused by DNA damage based on proliferation under RT and chemotherapy. However, the enhanced growth and migration potential in BTL2 as compared to BTL1 suggests that the observed differences might also reflect directed malignant progression. In contrast, the surprisingly low aggressiveness of BTL3 cells together with a comparable subclone in BTL1 cells suggest that dormant tumour cells from this or a comparable BTL1 subclone could have evaded the two surgeries as well as RT and chemotherapy. Correspondingly, Schmidt-Kittler *et al* (2003) have reported an early dissemination of single breast cancer cells with different chromosomal aberrations from the primary tumour to the surrounding healthy tissue. Absence of several alterations detected in the primary tumour suggested dissemination not only from the most advanced clone but also from pre-stages before immortalisation (Schmidt-Kittler *et al*, 2003). Alternatively, one might hypothesise that BTL3 is not derived from BTL1 but represents a second unrelated metachronous glioblastoma as has been reported before (Inda *et al*, 2003). However, several shared chromosomal alterations both in BTL cell lines as well as in the paired, paraffin-embedded tissue samples analysed (data not shown) strongly suggest in all cases derivation from a single founder clone that has developed to several genetically different subclones during tumour progression.

The first recurrence, represented by BTL2 cells, developed after ineffective chemotherapy and under RT. Consequently, one would expect enhanced chemoresistance of this and all following recurrences. Indeed, we detected moderately reduced sensitivity after a 72 h exposure of both BTL2 and BTL3 cells against the nitrosourea BCNU *in vitro*. To our surprise, the sensitivity against temozolomide, another alkylating agent, followed an inverse pattern revealing a continuous increase in temozolomide sensitivity from the primary tumour to the second recurrence. This was based on enhanced cytotoxic activity of temozolomide in both recurrence-derived cell lines with a particularly high cell death rate in BTL3 cells. At longer exposure times and higher cell density, BTL2 cells displayed the highest temozolomide-induced proliferation arrest, probably based on the more aggressive growth behaviour of these cells. Correspondingly, the patient responded to monotherapy with this agent after the first relapse. This might implicate that temozolomide as a strong mutagen in the absence of MGMT activity (Rabik *et al*, 2006) might have selected the less aggressive BTL3 cell subclone by preferentially killing the highly proliferative and not density-arrested BTL2 cell population. This is especially notable as temozolomide treatment was recently shown to significantly prolong overall survival of glioblastoma patients (Stupp *et al*., 2005) and thus has largely replaced nitrosoureas in the treatment of glioma (van den Bent *et al*, 2006).

With respect to temozolomide response, expression of the repair enzyme MGMT was suggested as the major resistance factor (Gerson, 2002) and lack of MGMT expression in gliomas is mainly based on gene promoter methylation (Silber *et al*, 1998; Esteller *et al*, 1999, 2000). Recently, promoter methylation was shown to represent an independent prognostic factor in glioblastoma patients (Hegi *et al*, 2005) and to be associated with enhanced overall survival after treatment with RT/temozolomide (Hegi *et al*, 2004). In our cell model, a persistent methylation of the MGMT gene promoter, even after application of temozolomide, has been detected in all three BTL cell lines, resulting in a complete lack of MGMT protein expression. Correspondingly, all BTL cell lines were sensitive to temozolomide, however, to a markedly different extent. Moreover, in the 72 h exposure vitality assay an inverse correlation of BCNU and temozolomide responsiveness was surprisingly observed. However, it has to be mentioned that at long BCNU exposure times (5 days), IC_50_ values for all three BTL cell lines averaged out at about 60–80 *μ*M (data not shown) whereas differences in case of temozolomide still were highly significant. As all three BTL sublines did not express MGMT, other resistance factors must underlie these observations. It has to be kept in mind that BCNU and CCNU, as chloroethylating agents, induce intra- and interstrand DNA crosslinks, whereas methylating agents, like temozolomide, primarily lead to O^6^-methylguanine adducts (Aquilina *et al*, 1994). Several recent observations suggest that resistance of glioblastomas to alkylating agents seems to follow a more complex pattern than simple dependence on MGMT levels. Thus, for example, the cellular p53 status and consequently inducibility of p21 by alkylating agents (Bocangel *et al*, 2002) as well as the base-excision repair capacity (Trivedi *et al*, 2005) were shown to influence alkylating agent response. Moreover, cells with mutated mismatch repair genes were particularly resistant to temozolomide, but not to chloroethylating agents (Gerson, 2002; Liu *et al*, 1996). In addition to repair mechanisms, also signals regulating cell death and survival pathways were implicated to regulate temozolomide response (Bredel *et al*, 2006).

When comparing alkylating agents with several other chemotherapeutics, an increased response in the recurrence-derived BTL cell lines was also observed in anthracyclines, whereas all cells were persistently unresponsive to VP-16, CDDP, and bleomycin. High-intrinsic chemoresistance of glioma cells has been reported by us and other groups involving overexpression of known drug-resistance proteins like P-gp, MRP1, and MVP (Berger *et al*, 2001; Bredel, 2001; Spiegl-Kreinecker *et al*, 2002). In our cell model, we found very low levels of mdr1 mRNA without detectable P-gp, persistent expression of MRP1, and reduction of MVP expression especially in BTL3 cells. In a previous study, we observed correlation between MVP expression of glioblastoma cells and resistance to anthracyclines (Berger *et al*, 2001), which suggests a contribution of MVP at least to doxorubicin hypersensitivity of BTL3 cells. Additionally, EGFR-transmitted signals are known to contribute to apoptosis resistance of glioblastoma cells (Shinojima *et al*, 2003) leading, for example, to CDDP insensitivity (Nagane *et al*, 1998). Moreover, response of xenografted gliomas to different alkylating agents was attenuated by EGFR amplification (Leuraud *et al*, 2004). Thus, the loss of EGFR amplification in BTL3 cells might also contribute to reduced chemoresistance.

From the clinical point of view, our data, although derived from only one cell model and a very limited number of cases, corroborate several clinical reports that have demonstrated activity of temozolomide in patients with recurring astrocytic brain tumours (for review see van den Bent *et al*, 2006). For example, a recent study reported a 43% response rate for salvage therapy with temozolomide for prior temozolomide responders at recurrence (Franceschi *et al*, 2005). In conclusion, we suggest that application of temozolomide represents a feasible strategy for glioblastoma treatment especially in patients with methylated MGMT promoter even at repeated recurrence and following chemo- and/or RT.

## Figures and Tables

**Table 1:** Course of disease of the BTL patient

**Month**	**0**	**1**	**2**	**3**	**4**	**5**	**6**	**7**	**8**	**9**	**10**	**11**	**12**	**13**	**14**	**15**	**16**	**17**	**18**	**19**	**20**	**21**	**22**	**23**	**24**	**25**	**26**	**27**
	OP	CCNU	RT	OP	2.Temo	3.Temo	4.Temo	5.Temo	6.Temo	7.Temo	Stable								MRI			MRI						Death
	(BTL1^a^)	1cycle		(BTL2^a^)			MRI			MRI	Disease								prog. OP			prog.						
				1.Temo			Regress			Regress									(BTL3^a^)									

a Establishment of the indicated BTL cell cultures from the respective surgery (OP) specimens.

CCNU, lomustine; RT, radiotherapy; Temo, temozolomide; prog, tumor progression.

**Table 3:** Chromosomal changes, therapy sensitivity, and overall survival in glioblastoma primary tumours (PT) and corresponding recurrences (Rec)

	**Number of changes^a^**			**Response to therapy^b^**			**OS**
**Case**	**PT**	**Rec 1**	**Rec 2**	**PT**	**Rec 1**	**Rec 2**	**Months**
GL1	9/1	12.5/2	18/3	—	—	—	21
GL2	9.3/3	12/5		—	—		17,5
GL3	3.7/3	17.5/2		—	—		14.5
GL4	24/1	n.a.^c^	14/1	+	n.t.^e^	n.t.	39
GL5	5.5/2	4.7/3		—	+		26 a.^d^
BTL	n.a.	n.a.	n.a.	—	+	n.t.	27

a Chromosomal aberrations were determined by CGH and chromosome arms with significant changes counted. Data are given as mean changes/microdissected areas analysed.

b Therapy of TMZ or CCNU was scored *in vivo* as + when the patient showed partial response or stable disease.

c Not analysed;

d Alive;

e No therapy.

**Table 2:** Chemosensitivity of the investigated cell lines

	**Cytotoxicity (IC_50_)^a^**							
**Cell line**	**TMZ (*μ*M)**	**BCNU (*μ*M)**	**DOX (nM)**	**L-DOX (nM)**	**DM (ng ml^−1^)**	**VP-16 (*μ*M)**	**CDDP (*μ*M)**	**BLEO (*μ*g ml^−1^)**
BTL-1	1225.0	141.10	200.00	83.30	150.00	>30.00	>10.00	>15.00
BTL-2	895.0	211.80	99.20	116.10	54.60	>30.00	>10.00	>15.00
BTL-3	367.6	287.00	39.30	9.30	82.20	>30.00	>10.00	>15.00

a Data are expressed as IC_50_ values (means of three experiments performed in triplicate) of the tested drugs (temozolomide, TMZ, carmustine, BCNU; doxorubicin, DOX; liposomal doxorubicin, L-DOX; daunomycin, DM; etoposide, VP-16; cisplatin, CDDP; bleomycin, BLEO).
